# Psilocybin in pharmacotherapy of obsessive-compulsive disorder

**DOI:** 10.1007/s43440-024-00633-1

**Published:** 2024-08-01

**Authors:** Maja Owe-Larsson, Katarzyna Kamińska, Barbara Buchalska, Dagmara Mirowska-Guzel, Agnieszka Cudnoch-Jędrzejewska

**Affiliations:** 1 Laboratory of Center for Preclinical Research, Department of Experimental and Clinical Physiology, Medical University of Warsaw, Banacha 1B, Warszawa, 02-097 Poland; 2 Department of Experimental and Clinical Pharmacology, Medical University of Warsaw, Warszawa, Poland

**Keywords:** Psilocybin, Psilocin, Obsessive-compulsive disorder, Serotonin

## Abstract

Obsessive-compulsive disorder (OCD) is a chronic mental disease that affects approximately 2% of the population. Obsessions and compulsions are troublesome for patients and may disturb their everyday activities. The pathogenesis of this disease is still not fully elucidated, but dysfunctions of serotonin-, dopamine- and glutamate-mediated neurotransmission together with early maladaptive schemas seem of importance. Pharmacological treatment includes drugs affecting the serotoninergic, dopaminergic, and glutamatergic systems, such as selective serotonin reuptake inhibitors (SSRIs). Providing that up to 40% of patients with OCD are resistant to the currently available medications, there is a need for novel and effective therapies. Recent discoveries suggest that psilocybin, a non-physically addictive psychoactive substance, may ameliorate disease symptoms. When used in appropriate doses and under strict clinical control, psilocybin appears as a valuable treatment for OCD. This narrative article provides a thorough overview of OCD’s etiology, current treatment options, and the emerging evidence supporting psilocybin’s efficacy in managing OCD symptoms.

## Introduction

Obsessive-compulsive disorder (OCD) is a chronic mental illness that affects approximately 1.6–2.3% of the human population [1]. The course of the disease is troublesome for patients, and, in its severe form, profoundly disturbs their daily activity. Usually, OCD is diagnosed early in life, before adulthood. Patients frequently experience obsessions, defined as recurring thoughts, impulses, needs, and/or compulsions - repeated, forced, and annoying performance of specific activities [1–3]. In light of available research, reduction of OCD symptoms can be achieved by modulation of the activity of serotonin- (5-HT), dopamine- and glutamate-mediated neurotransmission systems. However, approximately 40% of patients do not respond properly to the currently available drugs [1]. Hence, there is a need for new, potentially more effective therapeutic methods.

Psychedelics represent hallucinogens that produce false perceptions of non-existing objects or events involving sight, sound, smell, touch, and taste, known as hallucinations. Hallucinogenic substances include two groups of drugs, dissociative - such as dextromethorphan, ketamine, and phencyclidine, and classic - such as serotonergic and dopaminergic hallucinogens [4]. Recent years have brought a surge of data on the clinical use of psychedelic substances, in the therapy of neuropsychiatric conditions. The potential therapeutic applications include depressive symptoms [5, 6] post-traumatic stress disorder [7], substance addiction, pain treatment, and other [4, 8].

Psilocybin, a serotonergic psychedelic substance, induces antidepressant and anxiolytic effects in patients with endogenous depression or other depressive disorders. Improvements in mood and well-being, as well as increased overall life satisfaction, were observed in depressive patients for up to six months after treatment [9]. Over 100 species of mushrooms, mostly from the genus *Psilocybe*, commonly referred to as “magic mushrooms” or “hallucinogenic mushrooms”, contain psilocybin [10]. After oral intake, psilocybin is converted to its active metabolite, psilocin, in the intestinal mucosa. Both compounds act as agonists or partial agonists of G-protein coupled serotonin (5-HT) receptors, primarily of the inhibitory 5-HT_1A_ and excitatory 5-HT_2A/C_ types. Hallucinogenic actions of psilocybin and its metabolite seem to be mediated mostly through 5-HT_2A_ receptors [11–13]. Thus, it was hypothesized that psilocybin may also have a therapeutic potential in OCD. So far, the results of one completed clinical trial and several case reports indicate that psilocybin indeed ameliorates symptoms of this disease [14–17].

This article provides a thorough overview of OCD’s etiology, current treatment options, and the emerging evidence supporting psilocybin’s efficacy in managing OCD symptoms.

## Methods

A comprehensive review of publications, including surveys and systematic reviews, was performed to analyze the role of psilocybin in the management of OCD. Experimental and clinical studies were included, as well as clinical trial data. Articles presenting the putative mechanism of action of psilocybin, and its adverse effects in the context of the pathobiology of OCD were discussed. Scopus and PubMed databases were used to retrieve the articles with the following search terms: psilocybin, obsessive-compulsive disorder, psychedelic. The clinical trial database was also searched.

### Psilocybin– pharmacological targets

Psilocybin belongs to the group of psychedelics, acting through serotonergic receptors and thus influencing consciousness. Chemically, psilocybin is a tryptamine that contains an indole ring. The location of the methyl group at the C4 and C5 carbons is characteristic of psilocybin and psilocin, its active metabolite [18]. Psilocin is formed after oral administration of psilocybin by its dephosphorylation in the intestinal mucosa [19]. Psilocybin can be obtained from hallucinogenic psilocybin mushrooms, which have been used by humans for over 3,500 years. The active substance was isolated in 1958 by Albert Hoffmann and was exploited in experimental and clinical research for a short time. Studies on the usefulness of psilocybin largely ceased as a result of the abuse of psychedelics in the 1960s [20]. At that time, in the American legal act - the Controlled Substances Act of 1971 - psilocybin was classified as a Group I agent [12].

Psychedelics, including psilocybin, act on different targets in the serotonergic system. Their primary mechanism of action involves the stimulation of metabotropic 5-HT_2A_ and 5-HT_2C_ receptors, with subsequent activation of the G_α_ protein, and stimulation of the inhibitory 5-HT_1A_ and 5-HT _1D_ receptors, linked with the G_i/o_ protein [18]. The G_α_ protein increases the synthesis of cAMP (cyclic adenosine monophosphate) and stimulates a variety of intracellular processes, while G_i/o_ inhibits the formation of cAMP. Other targets of psilocybin and psilocin include 5-HT_1B_, 5-HT_1E_, 5-HT_2B_, 5-HT_5A_, 5-HT_6_, 5-HT_7_, D1, and D3 receptors. Both substances also display a weak affinity towards imidazoline, adrenergic-α_2A_, -α_2B_, -α_2C_ receptors, and the SERT transporter [21, 22]. Furthermore, psilocybin enhances intracellular signal transmission by a direct influence on the further steps of signaling cascades, including the early growth response proteins 1 and 2 (EGR1 and EGR2), involved in the modulation of synaptic plasticity and neuronal activity [23].

Psilocybin inhibits the default-mode network (DMN), which is active during the awareness period, but only under calm conditions. Yet, its activity declines when an individual is exposed to various forms of stimulation. This may explain the loss of identity after taking psilocybin. Anatomically, the resting-state network is located in the medial frontal and posterior cingulate cortex [18]. The effects of psilocybin, both psychedelic, and somatic, e.g. nausea, tremor, or cardiovascular effects (Table 1), are mediated mostly by psilocin [24]. Importantly, increased empathy, enhanced well-being, and improved creativity were observed already after the administration of a single dose of psilocybin (1 day after administration: *n* = 50, 7 days after: *n* = 22) [25].

**Table 1:** Central and somatic effects of psilocybin administration - based on Goel, Zilate, 2022, Irizarry et al., 2022 and Yerubandi et al., 2024 [23, 24, 140]

Central effects of psilocybin	Somatic effects of psilocybin
• euphoria • dissociation • hallucinations • hypnagogia • affective activation • dreams • introspection • mystical experiences • synesthesia • changed sense of time • anxiety	• increase/decrease in blood pressure • increase/decrease in heart rate • mydriasis • nausea • increased/weakened tendon reflexes • dysmetria • tremors • headache • dizziness

The use of psilocybin is practically not associated with the risk of addiction and was suggested as potentially beneficial in the treatment of addictive behaviors [23]. However, medical supervision over drug intake, adequate presentation, and awareness of the risks of medication, along with control of the mental and physical status of the patient can improve the safety of psilocybin for medical purposes [24, 26].

Psilocybin may also be combined with psychotherapy, especially CBT [29, 30]. Psychedelic-assisted psychotherapy is defined as the professionally supervised use of psilocybin and other substances: ketamine, MDMA, LSD, and ibogaine, as part of a psychological intervention [31]. A number of clinical trials reported that psilocybin used in depression and MDMA used in PTSD are safe and effective as add-on treatments [31, 32]. Another randomized controlled trial revealed that psilocybin augments autobiographical recollection, thus supporting the concept that psilocybin is a suitable substance in the course of psychotherapy. Two major benefits of psilocybin use are associated with the reversal of negative cognitive biases. Psilocybin may also aid the recall of salient memories [33].

### Obsessive-compulsive disorder

The Diagnostic and Statistical Manual of Mental Disorders (DSM)-5, developed by the American Psychiatric Association in 2013, defines OCD as a condition in which the patient’s obsessions cannot be explained by another mental disorder and are not the body’s response to psychoactive substances or possible illnesses, but, most of all, they are highly burdensome and time-consuming [1].

OCD is characterized by two main components: (a) obsessions, i.e. intrusive thoughts or images recurring without the patient’s will, causing strong feelings and fear, and (b) compulsions, i.e. actions intended to relieve anxiety caused by obsessions [34]. Usually, obsessions and compulsions are related to the fear of contamination - hence the forced and repetitive washing, soaping, cleaning of touched surfaces, avoiding touching objects, changing clothes multiple times during the day, etc. Obsessions can also involve the need for symmetry and order with a subsequent urge to arrange surrounding objects always in the same configuration. A disorderly environment brings profound fear and may even be perceived as a factor leading to the loss of health or life that may happen to patients and their relatives. Various rituals are performed to prevent potential harm, including e.g. checking dozens of times whether the oven is turned off, touching the wall of the room a certain number of times before leaving the house, repeatedly checking whether the entrance door is closed, etc. Unstoppable accumulation of even unnecessary items and a huge difficulty in getting rid of them (hoarding) is also frequent [35, 36]. The Yale-Brown Obsessive-Compulsive Scale (Y-BOCS) [37] divides manifestations of OCD into categories that can be assigned to five groups, presented in Table 2.

**Table 2:** OCD symptom groups and related obsessions and compulsions, according to Stein et al., 2019 [2]

Group of symptoms	Obsessions	Compulsions
Contamination symptoms	Concern, among others about dirt and germs	Washing, showering, cleaning
Harm-related symptoms	Concern about of incurring injury/accidents	Security checking
Unacceptability symptoms	Intrusive thoughts: aggressive, related to religion or sexuality	Mental rituals, prayers
Symmetry symptoms	Concern about the lack of symmetry	Arranging, straightening, repeating activities, counting
Hoarding symptoms	Concern about getting rid of things	Hoarding of items

### Etiology of OCD

**Fig. 1:**
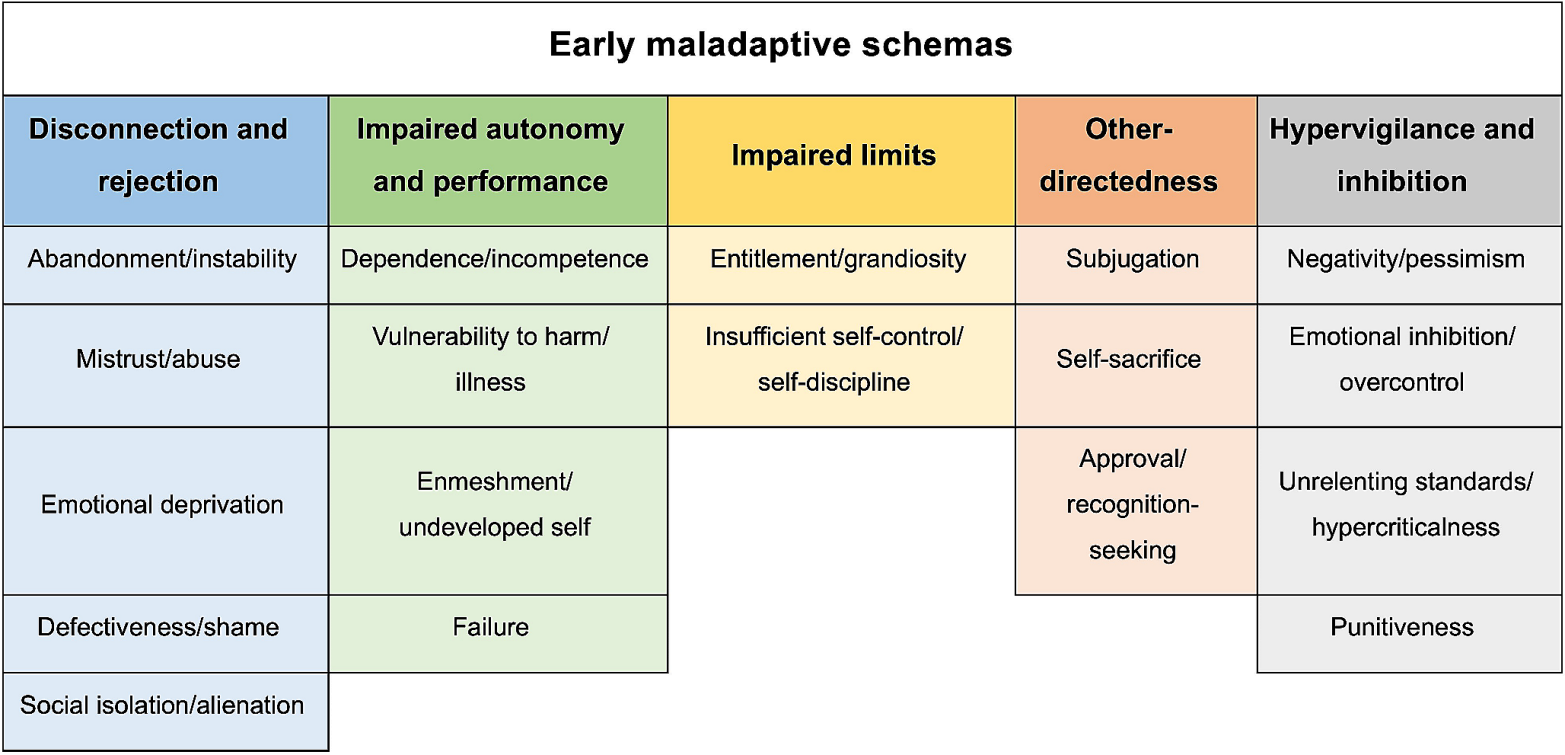
Early maladaptive schemas divided into 5 domains according to Young’s schema theory [39]

The development of OCD is multifactorial and involves psychological aspects and changes in the structure and functioning of the brain. In the emotional sphere of OCD development, 18 dysfunctional cognitive patterns (Early Maladaptive Schemas - EMS) have been described [38]. EMS, according to the schema theory of the American psychologist Jeffrey Young, are dysfunctional themes relating to the patient and his environment, developed in childhood as a response to unsatisfied emotional needs. They consist of memories, beliefs, bodily sensations, and emotions. EMS has been divided into 5 domains according to Young’s schema theory [39], see Fig. 1 for details.

OCD is hereditary in 27 to 45% of cases [1–3]. Studies in animal OCD models identified two possible risk genes for OCD: *SAPAP3* (SAP90/PSD95-associated protein 3) and *SLITRK5* (SLIT And NTRK Like Family Member 5) [40, 41]. Other risk genes include: *CHD8* (Chromodomain Helicase Animal Models for OCD Research DNA Binding Protein 8), associated also with autism, and *SCUBE1* (Signal Peptide, CUB Domain and EGF Like Domain Containing 1), crucial to the central nervous system development [42–44]. *SLC1A1* (solute carrier family 1 member 1), encoding the neuronal glutamate transporter 3 (EAAT3), and other gene variants influencing this gene’s expression were also linked to OCD [41, 45].

Altered neurotransmission, especially within cortico-subcortical loops, such as the cortico-striato-thalamo-cortical (CSTC) circuits, was implicated in the development of OCD [35, 46, 47]. CSTC circuits are responsible for motor and cognitive functions such as motivation and reward, response inhibition, and habitual behavior [35, 47, 48]. Projections from regions of the frontal cortex target the striatum, and then, via direct and indirect pathways through the basal ganglia, reach the thalamus and loop back to the cortex [49]. The direct pathway increases, while the indirect pathway decreases cortical excitation. OCD development is thought to be due to the direct pathway overactivity, and, in consequence, from hyperactivity of the CSTC circuits [35, 50].

Several structural alterations were detected in the brains of OCD patients. In a mega-analysis, an increased cerebellar volume and reduced sizes of the dorsomedial prefrontal cortex and the bilateral insular opercular region were detected in OCD patients (*n* = 412) compared to healthy subjects (*n* = 368)) [2, 51]. Furthermore, decreased cortical thickness in the superior and inferior frontal, precentral, posterior cingulate, middle temporal, inferior parietal, and precuneus gyri was described [52]. Larger volume of the putamen and decreased volume of the hippocampus were detected in adults with OCD. In child-onset OCD, thalamus volume was larger in untreated than in medicated children [53]. Increased volume of the white matter, especially in the anterior midline tracts, was also demonstrated [54]. Overall, these observations suggest neurodevelopmental changes in OCD [2].

Functional analyses demonstrated increased activity in response to symptom provocation or emotional processing in various brain regions: the orbitofrontal cortex, anterior cingulate cortex, ventral frontostriatal temporal region (e.g. hippocampus), ventromedial prefrontal cortex, middle temporal and left inferior occipital cortex, the bilateral amygdala and right putamen [55, 56]. In general, OCD patients display overactivation of brain areas engaged in arousal and habitual responses, whereas regions responsible for cognitive control are underactivated [57]. Caudate nucleus hyperactivation in OCD patients also implies increased habit formation [58]. The feeling of disgust is abnormally processed in the insula of contamination-related OCD patients [59].

**Fig. 2:**
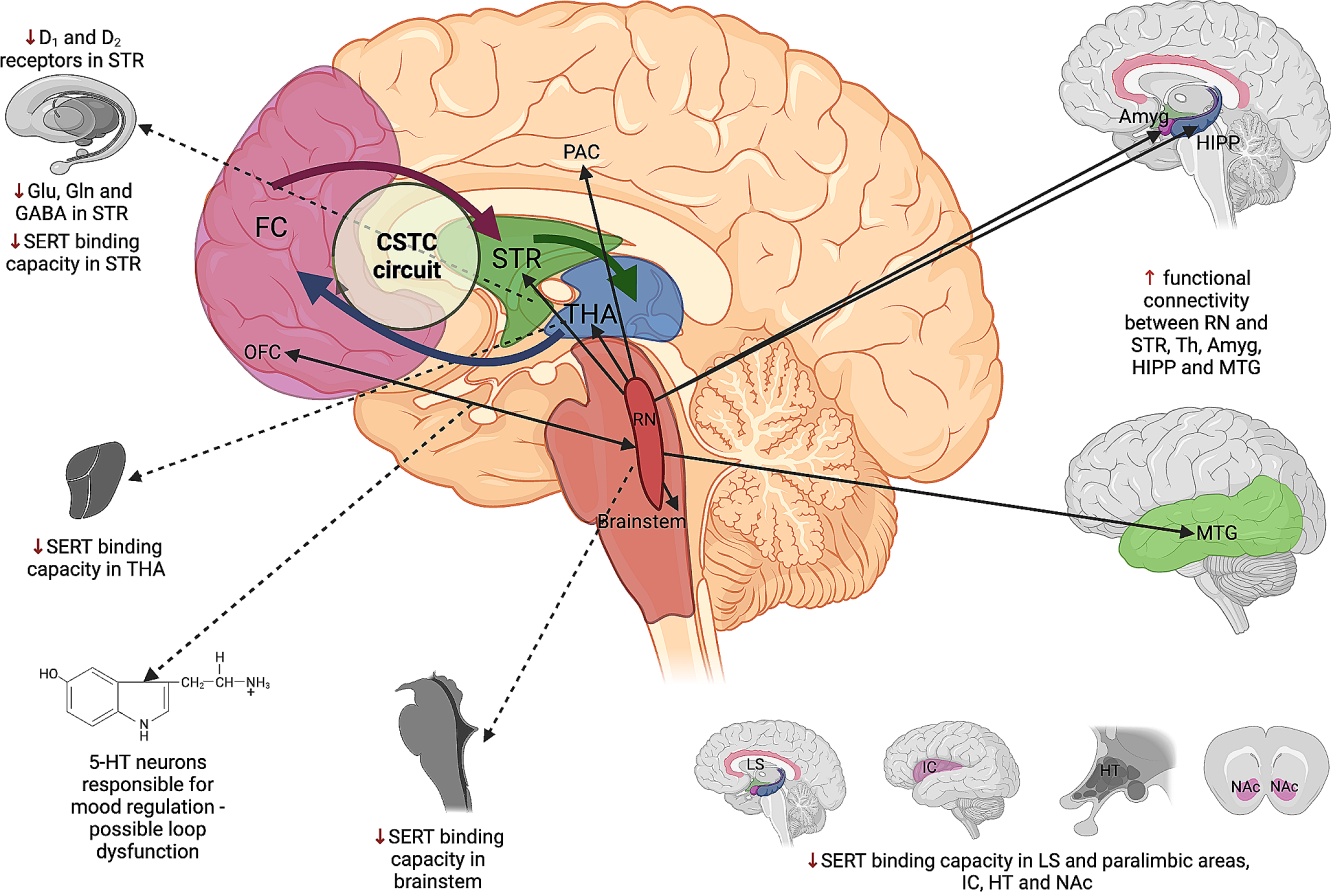
Neurobiological and molecular mechanisms of OCD pathogenesis focusing on serotoninergic disruptions. Amyg– amygdala, CSTC– cortico-striato-thalamic circuit, FC– frontal cortex, HIPP– hippocampus, HT– hypothalamus, IC– insular cortex, LS– limbic system, MTG– middle temporal gyrus, NAc– nucleus accumbens, OCD– obsessive-compulsive disorder, OFC– orbitofrontal cortex, PAC– paracingulate gyrus, PU– putamen, RN– raphe nuclei, STR– Striatum, THA– thalamus. Created with BioRender.com

At the molecular level, disturbances in serotonergic, dopaminergic, and glutamatergic transmission in the CSTC loops are associated with the development of OCD [2]. Proposed changes in signaling which are probably engaged in the development of OCD are depicted in Fig. 2 (Fig. 2).

The involvement of 5-HT in OCD pathogenesis is well substantiated. Serotoninergic neurons in the raphe nuclei (RN), dorsal (DRN), and median (MRN) project to nearly all parts of the brain. The RN controls emotions, learning, memory, locomotion, respiration, reproduction, or the sleep-wake cycle [60]. Reciprocal connections between 5-HT neurons of the DRN and the orbital cortices are thought to play a vital part in mood regulation, and thus, dysfunction of this loop was implied in OCD and depression [61]. Enhanced functional connectivity between the RN and temporal cortices, including the middle temporal and paracingulate gyri, amygdala, hippocampus, putamen, thalamus, and brain stem, was observed in patients with OCD [44]. Hyper-connectivity of the RN with the left middle temporal gyrus was also linked with the severity of OCD symptoms and was a significant predictor of response to 16-week SSRI therapy [62].

A significant decrease in mesencephalic serotonin transporter (SERT) binding was shown in 140 patients suffering from OCD [63]. Most studies demonstrated reduced brain SERT binding capacity in the midbrain, brainstem, thalamus, hypothalamus, limbic and paralimbic brain areas, nucleus accumbens, striatal regions, and insular cortex [64–66]. Others have reported normal [67] or even elevated [68] SERT binding. However, the groups of patients were much smaller in those single studies (*n* = 11 and *n* = 19, respectively). A negative correlation between OCD symptom severity and SERT availability was also detected [69–71]. Recent data have shown that SERT availability is reduced in late-onset but not in early-onset OCD [66]. Considering the above, selective serotonin reuptake inhibitors (SSRIs) are recommended as a first-line pharmacotherapy in moderate and severe OCD [72, 73].

The data on dopaminergic involvement in OCD are not fully clear. Dopamine agonists targeting the basal ganglia were shown to generate OCD-like behaviors in animals (quinpirole) and humans (methylphenidate and dextroamphetamine) [74, 75]. On the other hand, some studies have shown that increased cortical dopamine may improve symptoms of OCD [35, 76]. Striatal D2 receptor density was decreased, based on a retrospective analysis of positron emission tomography (PET) and single-photon emission tomography (SPECT) studies in 54 patients [63]. An increased dopamine transporter density was found in the basal ganglia of OCD patients [35, 77–79]. In general, dopaminergic activity seems to be enhanced in the course of OCD, and, indeed, antipsychotic drugs may improve OCD symptoms, especially compulsions and habitual behavior [35].

Glutamate plays a vital role in the physiological development of CSTC circuits and helps keep a balance between direct and indirect pathways [80]. Glutamatergic transmission disruptions in the CSTC circuit were proposed to be one of the pathogenetic mechanisms of OCD [81]. Lamotrigine, able to reduce the synaptic glutamate release, was shown to reduce both obsessions and compulsions. Topiramate, blocking kainate and α-amino-3-hydroxy-5-methyl-4-isoxazolepropionic acid (AMPA) glutamate receptors may also have a positive effect on OCD patients, though the data so far are inconclusive [82]. However, as lamotrigine and topiramate act also as voltage-gated sodium channel antagonists and the latter may stimulate γ-Aminobutyric acid a (GABAa) receptors, it is not fully clear whether their activity in OCD indeed results from changes in glutamate-mediated neurotransmission. Supporting the role of glutamate, presynaptic 5-HT_1B_ receptors are involved in controlling cortical and thalamic glutamatergic input to the basolateral amygdala, an area shown to be impaired in OCD [83]. Furthermore, several neuroimaging studies suggest a dysfunction in the postsynaptic glutamate signaling in the development of OCD [82]. Also, a sequence variation in *SLC1A1*, encoding the neuronal glutamate transporter 3 (EAAT3), was linked with susceptibility to OCD, particularly in males [45].

### Treatment of obsessive-compulsive disorder

The first-line treatment of OCD is cognitive-behavioral psychotherapy [84]. The therapy is based on the assumption that people’s behaviors and emotions depend on the acquired pattern of response to the environment and encountered situations. Initially, a negative thought pattern causing unwanted emotions and behaviors is identified. Treatment focuses on amending the patient’s incorrect reception and analysis of the information from the environment. When inaccurate beliefs are identified, they can be replaced with more appropriate and favorable views [85]. The form of cognitive behavioral therapy (CBT) most commonly used for OCD is exposure and response/ritual prevention (E/RP) therapy [86]. Conscious confronting of obsessive thoughts and situations that provoke obsessions is essential to abandon the obsessive action, even if such a thought/need arises. E/RP psychotherapy significantly reduces symptoms and is even more effective than pharmacological treatment. However, the costs of therapy are high, treatment is not available to every patient, and some patients are apprehensive about therapeutically induced anxiety [84].

Pharmacological treatment of OCD remains a challenge. Furthermore, detailed studies of response to applied pharmacotherapy are complicated by the fact that placebo response seems to affect in part the outcome of clinical trials [87]. Currently, first-line therapy includes the administration of SSRIs such as fluoxetine, fluvoxamine, sertraline, paroxetine, and citalopram. SSRIs reduce the 5-HT reuptake into neurons as a result of inhibition of the 5-HT transporter– SERT [88, 89]. SSRIs are used in monotherapy in patients poorly responding to or not willing to receive psychotherapy [84]. However, almost half of the patients initially do not respond to SSRI treatment. In these situations, the dose of the drug is gradually increased, often with good results. If treatment continues to fail, switching to another SSRI or drugs with a different mechanism of action is advised [90]. Yet, in a clinical scenario, higher doses of serotoninergic drugs increase the risk of adverse effects, caused by increased 5-HT_1A_ and 5-HT_2A_ receptor activity, such as changes in mental status, neuromuscular and autonomic hyperactivity, known as the serotonin syndrome [91].

When first-line drugs are ineffective, second-line treatment is introduced. SSRIs can be either switched to another SSRI, to clomipramine (a tricyclic antidepressant, TCA), or to venlafaxine (a serotonin-norepinephrine dual reuptake inhibitor) [92–97]. Switching between SSRIs brings improvement in only about 20% of patients, and the use of TCAs is associated with numerous side effects, such as tachycardia, constipation, xerostomia, impaired liver function, and lower seizure threshold. Venlafaxine on the other hand, with a similar effectiveness profile as SSRIs, is much better tolerated than clomipramine [84].

Other drugs interacting with serotoninergic neurotransmission were evaluated in the treatment of OCD, however, their effectiveness was not confirmed clinically [98]. Buspirone, a 5-HT_1A_ receptor agonist, was added to SSRI treatment in two studies (*n* = 14 in both), but its effectiveness was not higher than placebo [99, 100]. Its addition to clomipramine was not found to be effective either [98, 101]. Pindolol, a non-selective beta-blocker and 5-HT_1A_ receptor antagonist, demonstrated a statistically insignificant trend toward reducing symptoms of OCD (data from randomized placebo-controlled trials) in a meta-analysis of clinical experimental data [102]. Ondansetron, an antiemetic 5-HT_3_ receptor antagonist, was effective even at low doses, as shown in a systematic review of five controlled studies [103]. Nevertheless, the clinical trial has not met its primary efficacy endpoint [90]. Mirtazapine, a α_2_ receptor agonist and 5-HT _2 A/C_ and 5-HT_3_ antagonist, may be beneficial in OCD treatment or may quicken the response to a concurrent SSRI, according to two controlled studies [104, 105]. Still, more research is needed on this topic [90].

Synergistic treatment in OCD therapy is also attempted, as well as administering drugs acting on other neurotransmitter systems, e.g., the dopaminergic system. Antipsychotics such as aripiprazole, haloperidol, and risperidone, turned out to be more effective than placebo in the treatment of OCD, according to a meta-analysis [106].

Recent years have brought an increasing number of studies exploiting the utility of glutamate antagonists in OCD. Two antiepileptic agents, lamotrigine and topiramate, have shown some promising results in OCD [82, 84]. Both drugs inhibit voltage-gated sodium channels and reduce glutamate outflow, however, it is not clear whether their effects in OCD are linked with the modulation of glutamate-mediated neurotransmission [82, 84].

Memantine is a non-competitive antagonist of the N-methyl-d-aspartic acid (NMDA) receptor ion channel, used in the treatment of Alzheimer’s disease It displayed efficacy as an add-on therapy in moderate to severe OCD, as revealed by two randomized clinical trials [107]. However, the methodology of these studies was questioned [90, 108]. Clinical studies with larger cohorts, properly randomized and with well-defined populations of OCD patients, should be performed.

The benefits associated with the use of ketamine and esketamine as rapid-onset antidepressants are well substantiated [109, 110]. Ketamine, a derivative of phencyclidine, belongs to the group of dissociative psychedelics and displays a high affinity for the phencyclidine site of the NMDA receptor. Inhibition of NMDA receptors on GABAergic interneurons leads to a surge of glutamate and increases neuronal activity in the prefrontal cortex at low doses [111]. The use of ketamine and esketamine seems promising also in the therapy of less severely affected or unmedicated patients with OCD [112]. Therapeutic effects develop rapidly, in contrast to e.g. SSRIs, and the drugs can be used for a limited period of time, which improves patient compliance [82, 112, 113].

### Psilocybin in the treatment of OCD

In the search for a more effective pharmacological OCD therapy, psilocybin appears as an interesting, novel alternative. Although still rather limited, the results of experimental research, clinical case reports, and data from one clinical trial consistently indicate that psilocybin may be of value in the therapy of OCD. At present, seven other clinical trials are underway to verify the therapeutic potential of psilocybin in OCD [114–120].

In animal models of OCD, psilocybin was shown to be effective already after a single administration. A structure-activity relationship study of some psilocin and psilocybin derivatives revealed that in the mouse model of this disease, based on 5-HT-evoked itching, psilocybin, and psilocin, as well as 1-methylpsilocybin, in the doses of 5 mg/kg i.p., reduced the number of scratching episodes. The effect was not linked with 5-HT_2C_ receptors, as the activity of the studied substances in the OCD model did not correlate with the receptor affinity [121]. Another animal model of OCD employs so-called “marble burying” which represents the compulsive behavior of animals. Oral administration of the pulverized mushroom *Psilocybe argentipes*, with a high content of psilocybin, significantly reduced the number of compulsive marble-burying episodes [122]. In this model, administration of a 5-HT_2_ antagonist did not alter the activity of psilocybin. Similarly, a recent study in mice, using the marble burying model, showed that neither the 5-HT_1A_ nor the 5-HT_2A_ or 5-HT_2C_ receptors mediate the therapeutic effects of psilocybin [123]. Other studies also demonstrated that the reduction of digging evoked by psilocybin (2 mg/kg) was not reversed by the blockade of 5-HT_2A_ or 5-HT_2C_. Thus, an alternative mechanism seems to underlie the activity of psilocybin [124]. Its beneficial effects in OCD may result from targeting other serotoninergic receptors, interaction with the SERT transporter, or, possibly, with the dopaminergic receptors D1 and D3.

The first clinical report on the positive effect of psilocybin in OCD dates back to 1959 [125]. Case reports presenting significant improvement of OCD symptoms in patients taking psilocybin or mushrooms containing psilocybin followed [14, 15, 126–128]. Regardless of the type of OCD, psilocybin intake significantly reduces or even completely abolishes symptoms. The duration of therapeutic effects in individual patients varied from 4 to 5 days to three weeks without OCD symptoms [14, 15, 127]. In a patient consuming psilocybin mushrooms for four years, symptoms resolved completely during the first two years of the use of this substance. However, later on, OCD symptoms began to reoccur and they gradually returned to baseline levels [128].

So far, one clinical trial addressing the effects of psilocybin on OCD has been completed, but it was conducted on a small group of patients. This double-blind study included nine individuals who received up to 4 doses of psilocybin, at intervals of at least a week. Doses were ascending, from low (100 µg/kg) and medium (200 µg/kg) to high (300 µg/kg). In addition, a very low dose (25 µg/kg) was administered to patients at a random timepoint after the first dose, with the appropriate intervals kept.

The severity of OCD symptoms was evaluated at 0, 4, 8, and 24 h using the Y-BOCS scale. Symptom intensity decreased in all patients, by 23–100%. For most patients, improvement was still present at the 24-hour assessment. No significant adverse effects were observed, apart from a transient increase in blood pressure detected in one patient [16].

There are currently seven clinical trials concerning this topic underway. The study “Psilocybin for Treatment of Obsessive Compulsive Disorder (PSILOCD)” is no longer recruiting new participants [115]. Three other studies (“Efficacy of Psilocybin in OCD: a Double-Blind, Placebo-Controlled Study”, “Effects of Psilocybin in Obsessive Compulsive Disorder” and “PsilOCD: A Pharmacological-Challenge Feasibility Study”) are still recruiting patients [114, 116, 117]. For the first of them, a protocol describing the plan for carrying out research procedures has already been published. The study involves 30 adults who have failed standard treatment at least once and is randomized, double-blind, placebo-controlled (niacin), and non-crossover [129]. A protocol is also available for “PsilOCD: A Pharmacological-Challenge Feasibility Study”, which is a randomized, waitlist-controlled trial with blinded ratings [130]. The studies “Evaluating the Feasibility, Safety and Efficacy of Psychotherapy Assisted Psilocybin for Treatment of Severe OCD”, “Effects of Repeated Psilocybin Dosing in OCD” and “Feasibility, Clinical Effects, and Safety of Psilocybin-assisted Psychotherapy for Treatment-resistant OCD” have not yet started recruiting participants [118–120]. Detailed data regarding these studies are provided in Table 3. A case report describing one of the participants (trial NCT03356483) has already been published, revealing a significant reduction of OCD symptoms after treatment [17].

**Table 3:** Data describing clinical trials on psilocybin and OCD currently registered at ClinicalTrials.gov (Access: 2024, April 15)

Trial number at ClinicalTrials.gov	Study title	Status	Estimated date of completion of the study	Intervention	Estimated number of participants
NCT03300947	Psilocybin for Treatment of Obsessive Compulsive Disorder (PSILOCD)	Active, recruitment completed	May 30, 2023	psilocybin (100 µg/kg) or psilocybin (300 µg/kg) or lorazepam 1 mg	*n* = 15
NCT04882839	Evaluating the Feasibility, Safety and Efficacy of Psychotherapy Assisted Psilocybin for Treatment of Severe OCD	Before recruitment	December 1, 2025	combination of psychotherapy with psilocybin (1st session − 10 mg/70kg, 2nd session − 30 mg/70kg)	*n* = 15
NCT03356483	Efficacy of Psilocybin in OCD: a Double-Blind, Placebo-Controlled Study.	Recruitment ongoing	December 15, 2024	psilocybin (0.25 mg/kg) or niacin (250 mg)	*n* = 30
NCT05370911	Effects of Repeated Psilocybin Dosing in OCD	Before recruitment	August 2027	psilocybin (1st dose − 25 mg, 2nd dose − 25 mg or 30 mg)	*n* = 30
NCT05546658	Effects of Psilocybin in Obsessive Compulsive Disorder	Recruitment ongoing	September 12, 2026	psilocybin (1st session − 20 mg, 2nd session − 30 mg)	*n* = 30
NCT06299319	Feasibility, Clinical Effects, and Safety of Psilocybin-assisted Psychotherapy for Treatment-resistant OCD	Before recruitment	September 2024	psilocybin (25 mg)	*n* = 10
NCT06258031	PsilOCD: A Pharmacological-Challenge Feasibility Study	Recruitment ongoing	July 30, 2024	psilocybin (up to 10 mg, 2 occasions)	*n* = 20

It is noteworthy that psilocybin and psychedelics manifest therapeutic effects already after a single administration. Neurobiological data indicate that increased resting-state functional connectivity (RSFC) was observed within the DMN after discontinuation of treatment in patients with depression [131]. Increased ventromedial prefrontal cortex-bilateral inferior lateral parietal cortex RSFC was predictive of treatment response at 5 weeks, similarly to decreased parahippocampal-prefrontal cortex RSFC [131]. Similarly, alterations in connectivity were observed after the administration of lysergic acid diethylamide (LSD) [132].

In terms of cellular targets, it was suggested that tolerance to stress is mediated by the postsynaptic 5-HT_1A_ receptor, whereas actively addressing a source of stress is mediated by the 5-HT_2A_ receptor and linked with enhanced plasticity. It was proposed that the 5-HT_1A_ receptor pathway is enhanced by the SSRIs, while the 5-HT_2A_ receptor pathway is enhanced by 5-HT_2A_ receptor-agonist psychedelics, such as psilocybin [133]. Effector pathways downstream of serotonin 5-HT_2A_ receptor activation influence, among others, the gene expression patterns [134]. Notably, immediate early genes involved in neuroplasticity, such as *egr1* and *cfos*, or brain-derived neurotrophic factor (BDNF) are rapidly activated after psilocybin [135]. Induced neuroplastic changes in the prefrontal cortex with subsequent functional and structural alterations may help to restore proper brain function [136]. It is feasible that similar changes contribute to the improvement of OCD symptoms following psilocybin administration.

Interestingly, psychedelic experiences were demonstrated to help restoration of individual values and to promote social and pro-environmental attitudes. They seem to be driven by psychological insights [137–139]. Such a transformative epiphanic experience may be one of the factors contributing to a possible therapeutic effect of psilocybin and psychedelics in OCD.

### Adverse effects of psilocybin therapy

A recent meta-analysis revealed that the acute adverse effects associated with the use of psilocybin include headache, nausea, anxiety, dizziness, and elevated blood pressure. However, they seemed to be tolerable and receded within 48 h [140]. Available experimental data do not provide clear information on the potential cardiovascular effects and toxicity of psychedelics, including psilocybin. The drug seems relatively safe when tested in healthy volunteers [141]. Nevertheless, the use of substances is contraindicated in patients with uncontrolled hypertension [142, 143].

Although considered a rather safe low-toxicity hallucinogen, psilocybin is not devoid of potentially harmful adverse effects. The so-called “bad trip”, or “terrible excursion”, a phenomenon that occurs after the abuse of a psychoactive substance, is one of the most harmful consequences of psilocybin intake. Psychotic symptoms such as hallucinations and delusions, feelings of derealization (a sense of change, distance, and/or unreality of the surrounding world) and depersonalization (an altered sense of oneself as a person), anxiety, or panic attacks may be potentially dangerous for the patient. A “bad trip” has been described as an extremely unpleasant physical experience and is also common after the intake of lysergic acid diethylamide (LSD) [24, 26].

Hallucinogen-induced perceptual disorder, consisting of chronic visual hallucinations: optical snow, changes in color perception, diplopia, or a perceptive dysfunction, e.g. tinnitus, is another serious complication of psilocybin use. Depersonalization and derealization were also described. Two forms of this perceptual disorder, chronic and short (flashbacks) are known; they may last from weeks, months, years, or even up to a lifetime [144, 145].

Self-mutilation and other self-destructive behaviors were also reported in people with mental illnesses taking hallucinogenic mushrooms. Therefore, an absolute contraindication for the use of psilocybin is the history of psychotic events, as well as active schizophrenia and bipolar disorder, also in first-degree relatives. Manic symptoms (extreme talkativeness, agitation, decreased need for sleep, racing thoughts) may also be exacerbated in patients taking psilocybin [24, 26, 143, 146].

In OCD, the administration of psilocybin may induce minor adverse effects. Single reports describe psychedelic visions, nausea, vomiting, and dizziness immediately after consumption of mushrooms (one patient) [127]. Unpleasant sensations such as increased anxiety and a four-hour feeling of dissociation were reported by another patient [14]. It is of interest that several OCD patients who abused other psychoactive substances (e.g. cocaine and/or amphetamines) experienced actual worsening of disease symptoms [126, 127].

Criteria for including patients in psilocybin pharmacotherapy were proposed recently. They are based on intrapersonal and interpersonal factors and have been added to the criteria already used in psychiatric diagnosis. The three most important areas in patient evaluation have been suggested - clinical picture, therapeutic alliance, and patient safety. Implementing such procedures aims to improve treatment safety, patient care, and therapy outcomes [147].

After the promising results of a small clinical trial and positive conclusions from case reports, the results of further ongoing studies should be closely followed. Their outcome will hopefully allow to unequivocally answer the question of whether psilocybin is worth implementing in the treatment of OCD.

### Concluding remarks

OCD is a heterogeneous disorder with various symptom dimensions and underlying psychopathological and biological mechanisms. According to Stein et al. [2], OCD can be categorized into different symptom groups: contamination, harm-related, unacceptability, symmetry, and hoarding symptoms. These subgroups may also differ in their response to traditional treatments and may present unique challenges in clinical management.

Application of Psilocybin in OCD Subgroups:

1. Symptom Dimensions and Treatment Response:

- Contamination and Harm-Related Symptoms: Patients with contamination or harm-related obsessions and compulsions might respond differently to serotonergic interventions compared to those with symmetry or hoarding symptoms. The psilocybin’s agonistic action on 5-HT2A receptors might be particularly beneficial for these subgroups, which are often resistant to SSRIs.
- Symmetry and Hoarding Symptoms: These subgroups might have different neurobiological underpinnings, such as altered cortico-striato-thalamo-cortical (CSTC) circuitry and dopamine dysregulation. Psilocybin affects multiple neurotransmitter systems, including serotonin and dopamine, which could potentially benefit these patients.

2. Comorbidities:

- Depression and Anxiety: Comorbid depression and anxiety are common in OCD patients and can complicate treatment. Psilocybin has shown promise in alleviating depressive symptoms and could offer dual benefits for patients with comorbid conditions.
- Substance Use Disorders: Given that psilocybin is non-addictive and has been proposed as a treatment for addictive behaviors, it could be a suitable option for OCD patients with comorbid substance use disorders.

3. Early Maladaptive Schemas (EMS):

Early Maladaptive Schemas (EMS) are dysfunctional cognitive patterns developed in response to unmet emotional needs during childhood. Psilocybin’s ability to induce introspection and alter cognitive patterns might help patients reframe these maladaptive schemas.

Clinical Application and Considerations:

1. Personalized Treatment:
  Given the heterogeneity of OCD, a personalized approach to treatment is essential. Clinicians should consider the specific symptom dimensions, comorbidities, and individual patient profiles when evaluating the potential use of psilocybin.
2. Clinical Trials and Subgroup Analysis:
  Future clinical trials should include subgroup analyses to determine the efficacy of psilocybin across different OCD symptom dimensions and comorbid conditions. This would help identify which subgroups benefit the most from psilocybin therapy.
3. Safety and Monitoring:
  Strict clinical supervision is vital when using psilocybin. For patients with comorbidities, particularly those with a history of psychotic disorders or bipolar disorder, careful monitoring is crucial to prevent adverse effects.
4. Combination Therapies:
  Psilocybin might be used in combination with other therapies, such as cognitive-behavioral therapy and other pharmacological agents. For example, combining psilocybin with exposure and response prevention (E/RP) therapy could enhance treatment outcomes.
5. Patient Education and Informed Consent:
  Educating patients about the potential benefits and risks of psilocybin therapy is vital. Informed consent should include a discussion on the current state of research, potential side effects, and the importance of medical supervision.

### Future directions

Although experimental data are increasing, the clinical research on psilocybin in OCD is still limited and the evidence base is relatively small. There is a need for large, prospective trials evaluating the usefulness of psilocybin in therapy of OCD. Yet, incorporating psilocybin into clinical practice requires a nuanced understanding of OCD’s heterogeneity. By considering the different symptom dimensions, comorbidities, and individual patient profiles, clinicians can better evaluate the potential benefits of psilocybin therapy. Future research should focus on subgroup analyses in clinical trials and the development of personalized treatment protocols to optimize outcomes for OCD patients.

### Limitations

We are aware that still relatively small evidence base including several experimental research articles, case studies and a small clinical trial is a limitation of our study. However, the available data consistently indicate that psilocybin is an interesting therapeutic option as a sole or add-on treatment for OCD patients. Large-scale randomized controlled trials and long-term studies on the safety and efficacy of psilocybin are needed to clarify the value of psilocybin in the management of OCD.

## Data Availability

Data sharing not applicable to this article as no datasets were generated or analysed during the current study.

## Abbreviations

5-HT: Serotonin
AMPA: α-amino-3-hydroxy-5-methyl-4-isoxazolepropionic acid
Amyg: Amygdala
BDNF: Brain-derived neurotrophic factor
cAMP: Cyclic adenosine monophosphate
cfos: C-fos proto-oncogene
CBT: Cognitive behavioral therapy
CHD8: Chromodomain helicase animal models for OCD research DNA binding protein 8
CSTC: Cortico-striato-thalamo-cortical
DMN: Default-mode network
DRN: Dorsal raphe nucleus
DSM-5: Diagnostic and Statistical Manual of Mental Disorders-5
E/RP: Exposure and response/ritual prevention
EAAT3: Neuronal glutamate transporter 3
EGR1: Early growth response proteins 1
EGR2: Early growth response proteins 2
EMS: Early Maladaptive Schemas
FC: Frontal cortex
GABA: γ-Aminobutyric acid
HIPP: Hippocampus
HT: Hypothalamus
IC: Insular cortex
LS: Limbic system
MRN: Median raphe nucleus
MTG: Middle temporal gyrus
NAc: Nucleus accumbens
NMDA: N-methyl-d-aspartic acid
OCD: Obsessive-compulsive disorder
OFC: Orbitofrontal cortex
PAC: Paracingulate gyrus
PET: Positron emission tomography
PU: Putamen
RN: Raphe nuclei
RSFC: Resting-state functional connectivity
SAPAP3: SAP90/PSD95-associated protein 3
SCUBE1: Signal peptide, CUB domain and EGF like domain containing 1
SERT: Serotonin transporter
SLC1A1: Solute carrier family 1 member 1
SLITRK5: SLIT And NTRK like family member 5
SPECT: Single-photon emission tomography
SSRIs: Selective serotonin reuptake inhibitors
STR: Striatum
TCA: Tricyclic antidepressant
THA: Thalamus
Y-BOCS: Yale-Brown Obsessive-Compulsive Scale
